# Baseline neutrophil-to-lymphocyte ratio as a predictive and prognostic biomarker in patients with metastatic castration-resistant prostate cancer treated with cabazitaxel versus abiraterone or enzalutamide in the CARD study

**DOI:** 10.1016/j.esmoop.2021.100241

**Published:** 2021-08-24

**Authors:** R. de Wit, C. Wülfing, D. Castellano, G. Kramer, J.-C. Eymard, C.N. Sternberg, K. Fizazi, B. Tombal, A. Bamias, J. Carles, R. Iacovelli, B. Melichar, Á. Sverrisdóttir, C. Theodore, S. Feyerabend, C. Helissey, M.C. Foster, A. Ozatilgan, C. Geffriaud-Ricouard, J. de Bono

**Affiliations:** 1 Department of Medical Oncology, Erasmus Medical Center, Rotterdam, the Netherlands; 2 Department of Urology, Asklepios Tumorzentrum, Hamburg, Germany; 3 Department of Medical Oncology, University Hospital 12 de Octubre, Madrid, Spain; 4 Department of Urology, Medical University of Vienna, Vienna, Austria; 5 Department of Medical Oncology, Institute Jean Godinot, Reims, France; 6 Division of Hematology and Medical Oncology, Englander Institute for Precision Medicine, Weill Cornell Medicine, New York, USA; 7 Department of Cancer Medicine, Institut Gustave Roussy, Villejuif, France; 8 University of Paris Saclay, Saint-Aubin, France; 9 Institut de Recherche Clinique, Université Catholique de Louvain, Louvain, Belgium; 10 Department of Clinical Therapeutics, Alexandra Hospital, National and Kapodistrian University of Athens, Athens, Greece; 11 Vall d’Hebron Institute of Oncology, Vall d’Hebron University Hospital, Barcelona, Spain; 12 Medical Oncology Unit, Azienda Ospedaliera Universitaria Integrata (AOUI), Verona, Italy; 13 Department of Medical Oncology, Fondazione Policlinico Agostino Gemelli IRCCS, Rome, Italy; 14 Department of Oncology, Palacky University Medical School and Teaching Hospital, Olomouc, Czech Republic; 15 Department of Oncology, Landspitali University Hospital, Reykjavik, Iceland; 16 Department of Oncology, Foch Hospital, Suresnes, France; 17 Studienpraxis Urologie, Nürtingen, Germany; 18 Hôpital d’Instruction des Armées Bégin, Saint Mandé, France; 19 Global Medical Oncology, Sanofi, Cambridge, USA; 20 Europe Medical Oncology, Sanofi, Paris, France; 21 Division of Clinical Studies, The Institute of Cancer Research, London, UK; 22 Prostate Targeted Therapy Group, Royal Marsden Hospital, London, UK

**Keywords:** metastatic castration-resistant prostate cancer, cabazitaxel, abiraterone, enzalutamide, neutrophil-to-lymphocyte ratio, prognostic factor

## Abstract

**Background:**

There is growing evidence that a high neutrophil-to-lymphocyte ratio (NLR) is associated with poor overall survival (OS) for patients with metastatic castration-resistant prostate cancer (mCRPC). In the CARD study (NCT02485691), cabazitaxel significantly improved radiographic progression-free survival (rPFS) and OS versus abiraterone or enzalutamide in patients with mCRPC previously treated with docetaxel and the alternative androgen-receptor-targeted agent (ARTA). Here, we investigated NLR as a biomarker.

**Patients and methods:**

CARD was a multicenter, open-label study that randomized patients with mCRPC to receive cabazitaxel (25 mg/m^2^ every 3 weeks) versus abiraterone (1000 mg/day) or enzalutamide (160 mg/day). The relationships between baseline NLR [< versus ≥ median (3.38)] and rPFS, OS, time to prostate-specific antigen progression, and prostate-specific antigen response to cabazitaxel versus ARTA were evaluated using Kaplan–Meier estimates. Multivariable Cox regression with stepwise selection of covariates was used to investigate the prognostic association between baseline NLR and OS.

**Results:**

The rPFS benefit with cabazitaxel versus ARTA was particularly marked in patients with high NLR {8.5 versus 2.8 months, respectively; hazard ratio (HR) 0.43 [95% confidence interval (CI) 0.27-0.67]; *P* < 0.0001}, compared with low NLR [7.5 versus 5.1 months, respectively; HR 0.69 (95% CI 0.45-1.06); *P* = 0.0860]. Higher NLR (continuous covariate, per 1 unit increase) independently associated with poor OS [HR 1.05 (95% CI 1.02-1.08); *P* = 0.0003]. For cabazitaxel, there was no OS difference between patients with high versus low NLR (15.3 versus 12.9 months, respectively; *P* = 0.7465). Patients receiving an ARTA with high NLR, however, had a worse OS versus those with low NLR (9.5 versus 13.3 months, respectively; *P* = 0.0608).

**Conclusions:**

High baseline NLR predicts poor outcomes with an ARTA in patients with mCRPC previously treated with docetaxel and the alternative ARTA. Conversely, the activity of cabazitaxel is retained irrespective of NLR.

## Highlights

• Baseline NLR was evaluated as a biomarker in patients with mCRPC treated with cabazitaxel versus abiraterone or enzalutamide.
• High baseline NLR predicted poor outcomes with abiraterone or enzalutamide in patients with mCRPC.
• Clinical benefit from cabazitaxel was retained in higher baseline NLR patients.

## Introduction

Life-extending therapeutic options for patients with metastatic castration-resistant prostate cancer (mCRPC) have expanded in recent years from docetaxel chemotherapy to include cabazitaxel, androgen-signaling-targeted inhibitors (abiraterone and enzalutamide), radium-223, and sipuleucel-T. More recently, poly (ADP-ribose) polymerase inhibitors (olaparib and rucaparib) have become available for patients with DNA damage repair abnormalities.^1^, ^2^, ^3^ Docetaxel and androgen-signaling-targeted inhibitors also significantly prolong overall survival (OS) in metastatic hormone-sensitive prostate cancer, in combination with androgen deprivation therapy (ADT).^1^^,^^3^ Apalutamide, enzalutamide, and darolutamide are also approved for non-mCRPC.^1^^,^^3^

Although there have been advancements in determining optimal sequences of the many available agents for advanced prostate cancer, some uncertainties remain. It is now clear that patients who progress on abiraterone respond poorly to enzalutamide, and vice versa. This is likely because androgen-signaling-targeted inhibitors target the same pathway and share common resistance mechanisms.^2^^,^^4^, ^5^, ^6^ Results of the CARD study also indicate that patients who have received docetaxel and progressed within 12 months on abiraterone or enzalutamide should receive cabazitaxel over a second alternative androgen-signaling-targeted inhibitor.^6^ Cabazitaxel was associated with improved radiographic progression-free survival (rPFS), OS, prostate-specific antigen (PSA) response, and tumor response versus abiraterone or enzalutamide.^6^

A deeper understanding of the patient and tumor characteristics impacting treatment response is necessary, however, to inform optimal use and timing of available therapies.^7^ Much research has focused on the development of accurate prognostic biomarkers beyond conventional indicators such as stage, PSA level, Gleason score, and metabolic factors.^8^ Tumor-promoting inflammation is an enabling characteristic that underlies several hallmarks of cancer.^9^ Inflammation plays a crucial role in tumor development and metastatic progression in many cancers, including prostate cancer.^10^^,^^11^ Inflammation can also affect immune surveillance and responses to therapy.^10^^,^^11^

The neutrophil-to-lymphocyte ratio (NLR), a measure of the absolute neutrophil count (ANC) divided by the absolute lymphocyte count (ALC), is a biomarker of this systemic inflammation that is easy and inexpensive to measure in daily practice. NLR is a biomarker that integrates assessment of systemic inflammation as well as lymphocyte counts that are associated with the adaptive immune response to disorders ranging from infections to cancer.^12^^,^^13^ High NLR is associated with poor OS in many non-metastatic and metastatic solid tumors.^14^ In addition, high NLR has been associated with an increased risk of many non-neoplastic disorders, and has been shown to be predictive of the risk of developing cardiovascular disease.^15^ There is also growing evidence that high NLR is associated with poor OS in mCRPC.^16^, ^17^, ^18^, ^19^, ^20^, ^21^, ^22^, ^23^, ^24^, ^25^, ^26^ Although NLR is a continuous variable and an optimal cut-off for high NLR is not yet defined, retrospective analyses of phase III randomized studies and several meta-analyses have identified elevated baseline NLR as a strong indicator of prognosis in mCRPC.^16^^,^^18^^,^^21^^,^^24^, ^25^, ^26^ A recent meta-analysis involving 32 studies and 21 949 participants with prostate cancer found that high baseline NLR, neutrophil, and monocyte counts predict worse OS.^24^ The significant association between NLR and survival was maintained in a subgroup analysis including patients with mCRPC only, with the authors concluding that NLR may be a predominant prognostic factor in patients with mCRPC.^24^

Prognostic models for OS for patients with mCRPC have been developed with applications in clinical practice and clinical trial design.^27^, ^28^, ^29^ These models include parameters such as performance status, lactate dehydrogenase (LDH), PSA, albumin, hemoglobin, alkaline phosphatase, and presence of bone metastases.^27^, ^28^, ^29^ In addition to these traditional variables and treatment, NLR (<2.5 versus ≥2.5) was identified as a novel independent prognostic factor in the minimally symptomatic mCRPC setting by a validated model developed using the phase III PREVAIL database.^29^ As the PREVAIL study evaluated the efficacy and safety of enzalutamide versus placebo in chemotherapy-naive patients with mCRPC, this model reflects current clinical practice and points toward a role for NLR in risk stratification at diagnosis.^29^

The aim of this analysis was to investigate NLR as a predictive and prognostic biomarker in patients enrolled in the CARD study. Analyses investigating the association between baseline NLR and rPFS were prespecified; however, the CARD study was not powered on these analyses.

## Methods

### Study design

CARD was a multicenter, multinational, open-label study designed to compare cabazitaxel with abiraterone or enzalutamide in patients with mCRPC previously treated with docetaxel who progressed on the alternative androgen-signaling-targeted inhibitor (abiraterone or enzalutamide). Overall, 255 patients were randomized 1 : 1 to receive cabazitaxel (25 mg/m^2^ every 3 weeks plus prednisone daily and granulocyte colony-stimulating factor) versus abiraterone (1000 mg plus prednisone daily) or enzalutamide (160 mg daily) until radiographic progression, unacceptable toxicity, start of subsequent treatment, or patient request to discontinue treatment. The full study design, eligibility criteria, and efficacy and safety results have been described previously.^6^ The study was conducted in accordance with the Declaration of Helsinki and Good Clinical Practice guidelines and all patients provided written informed consent. The study is registered with clinicaltrials.gov, number NCT02485691.

### Procedures and outcomes

Hematology testing, which included ANC, ALC, and the calculation of NLR (ANC divided by ALC), was part of routine monitoring during the CARD study. The primary outcome of the CARD study was imaging-based PFS (also known as rPFS), defined as the time from randomization to radiologic tumor progression (assessed using RECIST 1.1) or progression of bone lesions [assessed using the Prostate Cancer Clinical Trials Working Group (PCWG2) criteria], or death from any cause. Secondary outcomes included OS, PSA response, and time to PSA progression. OS was defined as the interval between the date of randomization and the date of death from any cause. PSA response was defined as a decline in serum PSA of ≥50% from baseline confirmed by a second measurement at least 3 weeks later, in patients with a baseline PSA of ≥2 ng/ml. Time to PSA progression was calculated as the time from randomization to first documented PSA progression, which was defined in patients with a decline in PSA from baseline as an increase of ≥25% (at least 2 ng/ml) from the lowest value, confirmed at least 3 weeks later. In patients without a decline in PSA from baseline, PSA progression was defined as an increase of ≥25% (at least 2 ng/ml) from the baseline value following 12 weeks of treatment, confirmed at least 3 weeks later.

### Statistical analysis

The CARD study was designed to have 80% power to detect a hazard ratio (HR) of 0.67 (cabazitaxel versus an androgen-signaling-targeted inhibitor) in the analysis of the primary endpoint (rPFS) with the use of a stratified log-rank test at a two-sided alpha level of 5%. Approximately 234 patients needed to be randomized to collect data on 196 events (achieved at the cut-off date of 27 March 2019). All analyses reported here were carried out using data obtained at this cut-off date. Analyses were carried out in the intention-to-treat population. Baseline NLR was a prespecified prognostic factor in the CARD study; however, the analyses reported here were not powered on this parameter. Analyses investigating the association between baseline NLR (< versus ≥ median) and rPFS were planned in the protocol (see Supplementary Figure S1, Tables S1-S3, available at https://doi.org/10.1016/j.esmoop.2021.100241). All other analyses were *post hoc*.

The treatment effects of cabazitaxel versus abiraterone or enzalutamide on OS, rPFS, PSA response, and time to PSA progression by NLR subgroup were investigated using Kaplan–Meier survival estimates. The prognostic association between baseline NLR (as a continuous variable) and OS was investigated using stratified multivariate Cox regression with stepwise selection of covariates, including adjustment for treatment arm. In total, 14 pre-planned prognostic factors were evaluated. Categorical covariates were M1 disease, visceral metastases, prior therapy with curative intent (radical prostatectomy or radiation therapy for localized disease), type of progression, and Gleason score 8-10 at diagnosis, and continuous covariates were hemoglobin, duration of first ADT, NLR, neutrophil counts, age, testosterone, and log_10_-transformed LDH, alkaline phosphatase, and PSA. The significance thresholds for entry into and removal from the model were 0.10 and 0.05, respectively.

Exploratory analyses were conducted using Receiver Operating Characteristic (ROC) curves to estimate the optimal NLR threshold for the prediction of OS. As there was no strong evidence from the ROC analyses suggesting an optimal threshold, the median NLR (3.38) was selected as the cut-off threshold.

Pre-specified analyses were conducted using SAS version 9.2 and all *post hoc* analyses were conducted using SAS version 9.4.

## Results

### Patient population

A total of 255 patients with mCRPC were randomized to receive cabazitaxel (*n* = 129) or a second androgen-signaling-targeted inhibitor (*n* = 126) (Supplementary Figure S1, available at https://doi.org/10.1016/j.esmoop.2021.100241). At the cut-off date, median follow-up was 9.2 months and median duration of treatment was 22.0 weeks (range 13.1-30.4 weeks) with cabazitaxel compared with 12.5 weeks (range 9.9-23.4 weeks) with abiraterone or enzalutamide. Patient demographics and clinical characteristics have been reported previously.^6^ Briefly, 31% of patients were aged ≥75 years, 18% had visceral metastases, and most patients had pain progression at randomization (69%; Table 1). Median baseline neutrophil and lymphocyte counts were 4.48 × 10^9^/l and 1.3 × 10^9^/l across treatment arms, respectively, and the median baseline NLR was 3.38. Overall, patients with high NLR at baseline had higher PSA and LDH values compared with patients with low NLR at baseline (Table 1).

**Table 1:** Baseline clinical characteristics and laboratory parameters of patients enrolled in CARD

Characteristic	Cabazitaxel		Abiraterone or enzalutamide		Total	
	Low NLR (*n* = 62)	High NLR (*n* = 63)	Low NLR (*n* = 61)	High NLR (*n* = 60)	Low NLR (*n* = 123)	High NLR (*n* = 123)
Mean age, years (SD)	70.5 (8.7)	68.7 (8.0)	67.8 (7.5)	71.4 (8.1)	69.2 (8.2)	70.0 (8.1)
Metastatic sites, *n* (%)						
Bone (±lymph nodes)	36 (58.1)	35 (55.6)	36 (59.0)	37 (61.7)	72 (58.5)	72 (58.5)
Lymph nodes	6 (9.7)	2 (3.2)	5 (8.2)	1 (1.7)	11 (8.9)	3 (2.4)
Liver or lung	11 (17.7)	10 (15.9)	8 (13.1)	15 (25.0)	19 (15.4)	25 (20.3)
Other	9 (14.5)	16 (25.4)	12 (19.7)	7 (11.7)	21 (17.1)	23 (18.7)
Mean PSA, ng/ml^a^	163.1	374.7	231.0	192.8	196.8	284.5
Mean neutrophil count, mm^3^	3770.1	6204.0	3845.6	5579.8	3807.5	5899.5
Mean hemoglobin, g/l	122.7	121.4	120.9	122.0	121.8	121.7
Mean LDH, IU/l^b^	291.5	367.5	307.4	372.1	299.4	369.7
Type of progression at trial entry, *n* (%)						
PSA only^c^	5 (8.1)	6 (9.5)	7 (11.5)	3 (5.0)	12 (9.8)	9 (7.3)
Radiologic progression^d^	8 (12.9)	14 (22.2)	6 (9.8)	9 (15.0)	14 (11.4)	23 (18.7)
Pain status	46 (74.2)	38 (60.3)	45 (73.8)	41 (68.3)	91 (74.0)	79 (64.2)
Missing	3 (4.8)	5 (7.9)	3 (4.9)	7 (11.7)	6 (4.9)	12 (9.8)
No metastatic (M1) disease at diagnosis, *n* (%)	42 (67.7)	35 (55.6)	28 (45.9)	33 (55.0)	70 (56.9)	68 (55.3)
Gleason score 8-10 at diagnosis, *n* (%)	34 (54.8)	36 (57.1)	43 (70.5)	36 (60.0)	77 (62.6)	72 (58.5)
Duration of first ADT <12 months, *n* (%)	26 (41.9)	29 (46.0)	32 (52.5)	24 (40.0)	58 (47.2)	53 (43.1)
Previous ARTA, *n* (%)						
Abiraterone	25 (40.3)	28 (44.4)	35 (57.4)	31 (51.7)	60 (48.8)	59 (48.0)
Enzalutamide	37 (59.7)	34 (54.0)	26 (42.6)	29 (48.3)	63 (51.2)	63 (51.2)
Missing	—	1 (1.6)	—	0	—	1 (0.8)
ARTA received after docetaxel, *n* (%)	37 (59.7)	39 (61.9)	41 (67.2)	35 (58.3)	78 (63.4)	74 (60.2)
Time from ARTA initiation to progression 0-6 months, *n* (%)	33 (53.2)	31 (49.2)	30 (49.2)	28 (46.7)	63 (51.2)	59 (48.0)

High/low NLR categories were based on the median NLR at baseline (3.38).

ADT, androgen deprivation therapy; ARTA, androgen-receptor-targeted agent; LDH, lactate dehydrogenase; NLR, neutrophil-to-lymphocyte ratio; PSA, prostate-specific antigen; SD, standard deviation.

a Three patients missing for cabazitaxel low NLR, two patients for cabazitaxel high NLR, three patients for ARTA low NLR.

b One patient missing for ARTA low NLR and one patient for ARTA high NLR.

c No radiologic progression and no pain.

d ±Rising PSA and no pain.

### Treatment exposure by NLR subgroup

The treatment durations and frequency of dose modifications associated with cabazitaxel and abiraterone/enzalutamide for each NLR subgroup are shown in Supplementary Table S1, available at https://doi.org/10.1016/j.esmoop.2021.100241. Cabazitaxel treatment exposure was generally similar across NLR subgroups; more patients with low NLR had an abiraterone or enzalutamide dose reduction (45.9%) compared with those with high NLR (28.3%; Supplementary Table S1, available at https://doi.org/10.1016/j.esmoop.2021.100241).

### Influence of baseline NLR on median rPFS

**Figure 1:**
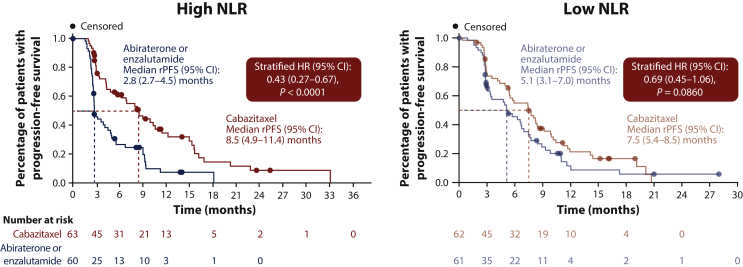
rPFS by baseline NLR. CI, confidence interval; HR, hazard ratio; NLR, neutrophil-to-lymphocyte ratio; rPFS, radiographic progression-free survival.

As previously reported, in the overall population, median rPFS [95% confidence interval (CI)] was 8.0 months (5.7-9.2 months) in patients receiving cabazitaxel compared with 3.7 months (95% CI 2.8-5.1 months) in patients receiving an androgen-signaling-targeted inhibitor.^6^ The benefit of cabazitaxel was particularly marked in patients with high baseline NLR. Indeed, in such patients, a median rPFS of 8.5 months (95% CI 4.9-11.4 months) was observed in patients receiving cabazitaxel compared with 2.8 months (95% CI 2.7-4.5 months) in patients receiving an androgen-signaling-targeted inhibitor [HR 0.43 (95% CI 0.27-0.67); *P* < 0.0001; Figure 1]. For patients with low baseline NLR, median rPFS was 7.5 months (95% CI 5.4-8.5 months) with cabazitaxel versus 5.1 months (95% CI 3.1-7.0 months) with abiraterone or enzalutamide [HR 0.69 (95% CI 0.45-1.06); *P* = 0.0860; Figure 1].

### Baseline NLR is associated with OS

In univariate analyses, with all arms combined, the following baseline factors were associated with worse OS: higher NLR, higher PSA values, lower hemoglobin values, higher LDH values, higher alkaline phosphatase values, and, as expected, higher neutrophil counts (Supplementary Table S2, available at https://doi.org/10.1016/j.esmoop.2021.100241).

In the final multivariate model, high NLR (as a continuous covariate) emerged as independently associated with worse OS. Other independent factors associated with poor OS were low hemoglobin and high baseline PSA levels (Supplementary Table S3, available at https://doi.org/10.1016/j.esmoop.2021.100241). In the presence of these factors, the survival benefit associated with cabazitaxel versus abiraterone or enzalutamide remained significant [HR 0.63 (95% CI 0.42-0.94); *P* = 0.022; Supplementary Table S3, available at https://doi.org/10.1016/j.esmoop.2021.100241].

**Figure 2:**
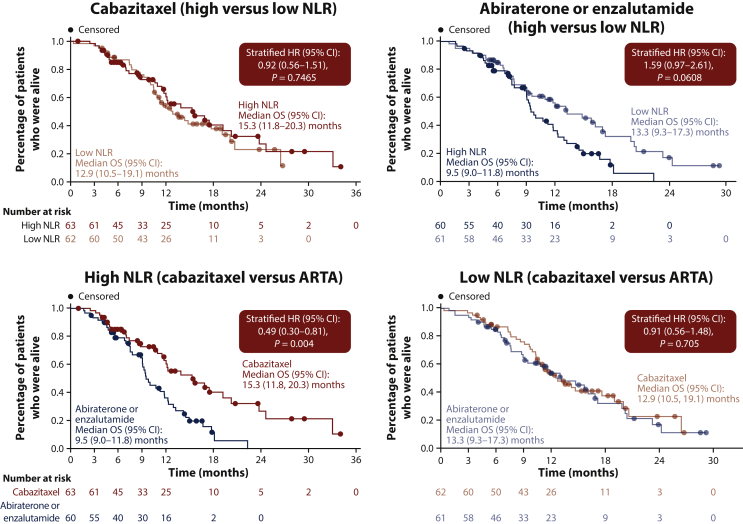
OS by baseline NLR. ARTA, androgen-receptor-targeted agent; CI, confidence interval; HR, hazard ratio; NLR, neutrophil-to-lymphocyte ratio; OS, overall survival.

As previously reported, median OS was 13.6 months with cabazitaxel and 11.0 months with abiraterone or enzalutamide [HR 0.64 (95% CI 0.46-0.89); *P* = 0.008].^6^ In patients treated with cabazitaxel, there was no significant OS difference between patients with a high NLR at baseline [15.3 months (95% CI 11.8-20.3 months)] and those with a low NLR at baseline [12.9 months (95% CI 10.5-19.1 months), *P* = 0.7465; Figure 2]. However, patients treated with abiraterone or enzalutamide with high NLR at baseline showed a numerically worse OS versus those with low NLR at baseline [9.5 months (95% CI 9.0-11.8 months) versus 13.3 months (95% CI 9.3-17.3 months); *P* = 0.0608; Figure 2]. These data may suggest that the OS benefit of cabazitaxel versus abiraterone or enzalutamide is particularly marked in patients with a high NLR at baseline [HR 0.49 (95% CI 0.30-0.81); *P* = 0.004; Figure 2] compared with those with low NLR at baseline [HR 0.91 (95% CI 0.56-1.48); *P* = 0.705; Figure 2].

### Influence of baseline NLR on time to PSA progression and PSA response

**Figure 3:**
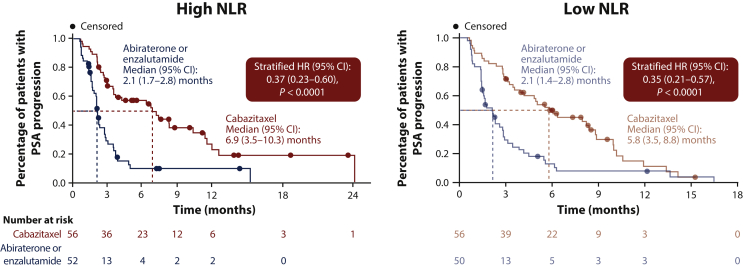
Time to PSA progression by baseline NLR. CI, confidence interval; HR, hazard ratio; NLR, neutrophil-to-lymphocyte ratio; PSA, prostate-specific antigen.

Cabazitaxel prolonged time to PSA progression versus abiraterone or enzalutamide, with the benefit of cabazitaxel particularly marked in patients with high baseline NLR. In patients with high baseline NLR, median time to PSA progression was 6.9 months (95% CI 3.5-10.3 months) with cabazitaxel versus 2.1 months (95% CI 1.7-2.8 months) with abiraterone or enzalutamide [HR 0.37 (95% CI 0.23-0.60); *P* < 0.0001; Figure 3]. Low baseline NLR was associated with a median time to PSA progression of 5.8 months (95% CI 3.5-8.8 months) with cabazitaxel versus 2.1 months (95% CI 1.4-2.8 months) with abiraterone or enzalutamide [HR 0.35 (95% CI 0.21-0.57); *P* < 0.0001; Figure 3].

**Figure 4:**
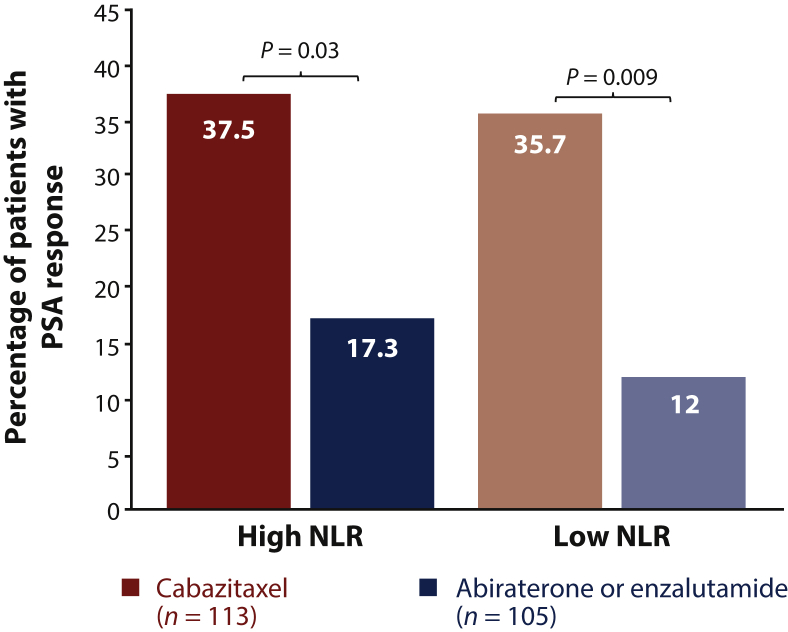
PSA response by baseline NLR. NLR, neutrophil-to-lymphocyte ratio; PSA, prostate-specific antigen.

As previously reported, PSA response was observed in 41/115 patients (36%) receiving cabazitaxel versus 15/111 (14%) receiving an androgen-signaling-targeted inhibitor in the overall population.^6^ PSA response with cabazitaxel was not influenced by patient baseline NLR (Figure 4). PSA response with abiraterone or enzalutamide remained low in patients with high NLR (17.3%) and low NLR (12%).

## Discussion

Although the prognostic value of baseline NLR has been the subject of debate across several malignancies, it is now accepted that baseline NLR is prognostic in mCRPC.^11^ A greater understanding of the predictive value of NLR, however, is necessary to guide decision making in the context of the many available treatment options. The CARD study established the superiority of cabazitaxel over abiraterone or enzalutamide in patients with mCRPC who had previously received docetaxel and an alternative androgen-signaling-targeted inhibitor.^6^ Evaluating the prognostic and predictive value of NLR in such a study was thus of great interest. The present findings may be summarized as follows: first, high NLR was prognostic when all arms were combined, but analysis by treatment arm showed that NLR was prognostic only in patients treated with abiraterone or enzalutamide, and not in those treated with cabazitaxel. Patients with high NLR at baseline who received abiraterone or enzalutamide had a 4-month lower median OS compared with patients with low NLR (9.5 versus 13.3 months), whereas patients treated with cabazitaxel with a high NLR had a numerically longer median OS than those with a low NLR (15.3 versus 12.9 months). Second, high NLR, compared with low NLR, was associated with reduced antitumor activity of abiraterone or enzalutamide in terms of rPFS and PSA response. Conversely, although cabazitaxel was superior to abiraterone or enzalutamide regardless of low or high NLR, the clinical antitumor activity of cabazitaxel appeared numerically greater in patients with high NLR.

This association between high NLR and poorer responses to androgen-signaling-targeted inhibitors seen in the CARD population is consistent with several retrospective studies.^17^^,^^19^^,^^20^^,^^22^^,^^23^ Leibowitz-Amit et al.^17^ first reported that NLR >5 was associated with a poor PSA response and shorter OS in two independent cohorts of patients treated with abiraterone at the Princess Margaret and Royal Marsden Cancer Centers. Other investigators have reported similar findings, either with abiraterone or enzalutamide.^19^^,^^20^^,^^22^^,^^23^ In a cohort of 193 patients with mCRPC treated with enzalutamide after docetaxel, patients with baseline NLR >3 had a median OS of 10.4 months, compared with 16.9 months in patients with baseline NLR ≤3.^19^ A *post hoc* analysis of the pivotal phase III COU-AA-302 study conducted in 1088 asymptomatic or minimally symptomatic chemotherapy-naive patients with mCRPC also identified high baseline NLR (>2.5) as a predictor of shorter OS, rPFS, and PSA-PFS in patients receiving abiraterone.^23^

In contrast, patients enrolled in CARD who received cabazitaxel consistently had improved clinical outcomes compared with patients who received an androgen-signaling-targeted inhibitor, regardless of baseline NLR. Patients with high baseline NLR had higher baseline ANC values compared with those with low baseline NLR; however, this was not associated with an increase in cabazitaxel dose intensity. To our knowledge, this is the first analysis to demonstrate that treatment with cabazitaxel may counteract the poor prognosis associated with high baseline NLR in patients with advanced prostate cancer.

Given that patients with mCRPC and high NLR have poor outcomes when treated with abiraterone or enzalutamide, this biomarker can be used to identify patients in whom treatment with a second androgen-signaling-targeted inhibitor should be avoided. Furthermore, cabazitaxel may counteract the adverse prognosis associated with high NLR, comparable with how trastuzumab reverses the poor prognosis associated with HER2 positivity.^30^ As NLR can be measured during a simple blood test, NLR status could be an easy and accessible way of determining the optimal next therapy in patients with mCRPC who progress on docetaxel and an initial androgen-signaling-targeted inhibitor.

In cancer patients, baseline NLR is likely a biomarker of systemic inflammation, which has emerged as a key mechanism for tumor progression.^11^ Although NLR is measured in the peripheral blood, it is the levels of leukocytes in the tumor microenvironment that are the crucial factor. However, it is possible that elevated baseline NLR reflects either a decreased lymphocyte count or an increased myeloid cell count, or both. There is increasing evidence that tumor-associated myeloid cells drive tumor progression through multiple mechanisms.^11^^,^^31^^,^^32^ Neutrophils can suppress CD8+ T-cell activation to facilitate metastasis.^33^ In cancer, neutrophils are also often associated with myeloid-derived suppressor cells (MDSCs), which also contribute to an immunosuppressive microenvironment.^32^ It has been shown that androgen deprivation leads to subsequent MDSC infiltration and interleukin-23 production, fostering mCRPC development.^34^ The findings that, in solid tumors and hematologic malignancies, grade ≥3 neutropenia or leucopenia during chemotherapy is consistently associated with improved OS, may also reflect the role of neutrophils in promoting cancer progression, although the effects of increased cytotoxic drug exposure should also be taken into consideration.^35^ In a randomized study comparing docetaxel with mitoxantrone in 228 Chinese patients with mCRPC, docetaxel was associated with a much greater OS benefit (an increase of 8.2 months) than observed in the randomized TAX327 study, conducted in 1006 Caucasian patients (an increase of 2.4 months).^36^^,^^37^ The increased OS benefit seen in the Chinese study was associated with an increased rate of grade ≥3 neutropenia (58% versus 32%).^36^^,^^37^ A *post hoc* analysis of the phase III TROPIC study of cabazitaxel versus mitoxantrone in 755 patients with mCRPC previously treated with docetaxel also found that patients with grade ≥3 neutropenia had significantly longer OS [HR 0.65 (95% CI 0.43-0.97)] and PFS [HR 0.63 (95% CI 0.42-0.95)] compared with patients without grade ≥3 neutropenia.^38^

There are several important limitations of the present analysis. Firstly, prior administration of corticosteroids that induce the release of neutrophils and suppress lymphocyte counts, thereby altering NLR, may represent a confounding factor.^21^ Secondly, the CARD study was powered for rPFS, but was not powered for the secondary endpoints and *post hoc* analyses reported here, due to the relatively small number of patients enrolled in CARD (*n* = 255). Third, patients in CARD progressed with prior abiraterone or enzalutamide within 12 months and results may therefore not be generalizable to patients progressing with androgen-signaling-targeted inhibitors beyond 12 months, although median time to progression with abiraterone or enzalutamide did not exceed 1 year in randomized studies conducted in chemotherapy-naive mCRPC.^4^^,^^5^ The results reported here also may not be generalizable to patients receiving an androgen-signaling-targeted inhibitor in non-mCRPC. Therefore, although the results suggest that patients with high NLR may have a greater benefit with cabazitaxel over a second androgen-signaling-targeted inhibitor, further validation of this is required. Finally, the median NLR value used in the present study may not represent the optimal cut-off value for defining those patients with poor outcomes. In this subanalysis, the median NLR (3.38) was used as the cut-off, as ROC did not identify an alternative optimal cut-off. As discussed, there is no clear consensus regarding what the optimal cut-off is, with other studies using cut-offs ranging from 2.5 to 5.^17^^,^^19^^,^^20^^,^^22^^,^^23^ Rationales for these cut-offs vary per study, although the reason is frequently based on the median value of the population or the median of an earlier study. The variability in cut-offs highlights a need for validation and identification of an optimal cut-off that can be used consistently in future studies. More research into risk stratification using this continuous variable as a biomarker is needed.

### Conclusions

In conclusion, the findings of this analysis show that high NLR at baseline associates with poor outcomes with abiraterone or enzalutamide, but does not appear to impact the antitumor activity of cabazitaxel. We hypothesize that high NLR reflects an immunosuppressive environment in which tumor-associated neutrophils and MDSCs promote tumor growth. Such an environment may induce resistance to abiraterone or enzalutamide, while cabazitaxel, by targeting the microtubule network of these neutrophils and myeloid cells, contributes to improving OS. Combined with the previously published survival and health-related quality of life benefits,^6^^,^^39^ the finding that cabazitaxel is superior to a second androgen-signaling-targeted inhibitor regardless of baseline NLR strengthens the support for the use of cabazitaxel over abiraterone or enzalutamide in this setting. Further studies are necessary to fully elucidate the underlying mechanisms, which should contribute to a better understanding of the role of the tumor microenvironment in prostate cancer.
