# Supplementary material for: Baseline neutrophil-to-lymphocyte ratio as a predictive and prognostic biomarker in patients with metastatic castration-resistant prostate cancer treated with cabazitaxel versus abiraterone or enzalutamide in the CARD study
Source: ESMO Open. 2021 Aug 24;6(5):100241. doi: 10.1016/j.esmoop.2021.100241 (PMC8390550; doi:10.1016/j.esmoop.2021.100241)
Supplement: Supplementary Figure and Tables [file mmc1.pdf]

Figure A.1. CONSORT diagram

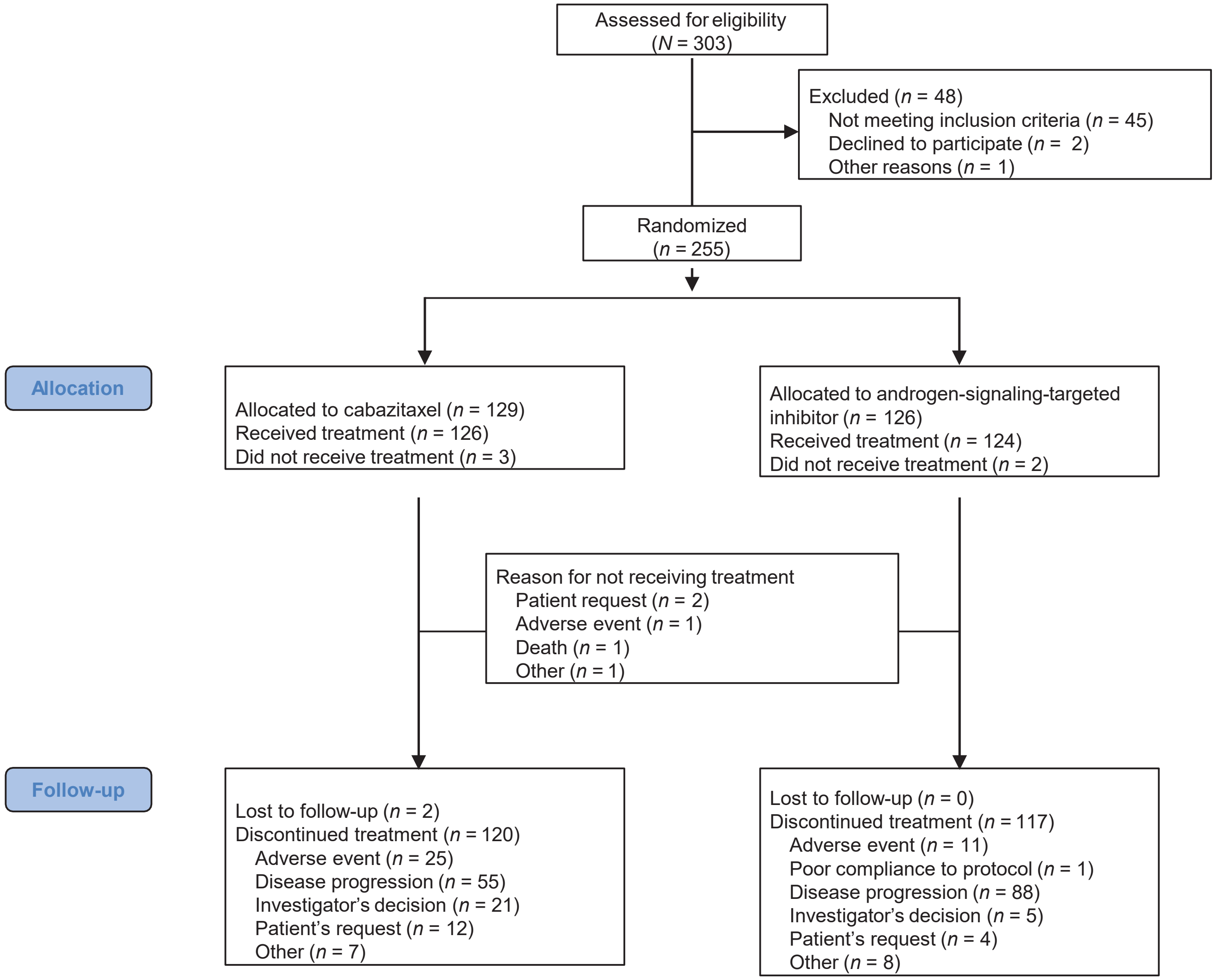

Supplementary tables

Table A.1. Treatment exposure by NLR subgroup

**Treatment**	**Cabazitaxel**		**Abiraterone or enzalutamide**	
	**Low NLR (*n* = 62)**	**High NLR (*n* = 63)**	**Low NLR (*n* = 61)**	**High NLR (*n* = 60)**
Treatment duration				
Median duration of treatment exposure, weeks (IQR)	22.9 (13.0–30.0)	22.0 (13.4–35.0)	15.1 (11.9–26.6)	12.1 (9.7–18.3)
Median number of cycles administered (IQR)	7.0 (4.0–10.0)	7.0 (4.0–11.0)	5.0 (4.0–9.0)	4.0 (3.0–6.0)
Patients with at least one dose reduction, n (%)	14 (22.6)	12 (19.0)	28 (45.9)	17 (28.3)
Number of dose reductions	17	14	36	25
Patients with at least one dose delay, n (%)	24 (38.7)	30 (47.6)		
Number of dose delays	38	38	-	-
Patients with at least one dose interruption, n (%)	5 (8.1)	2 (3.2)	-	-
Number of dose interruptions	5	2		

Abbreviations: IQR, interquartile range; NLR, neutrophil-to-lymphocyte ratio.

Table A.2. *Univariate Cox regression analysis of OS*

**Baseline risk factor**	**HR (95% CI)**	***P* value**
Treatment (cabazitaxel vs abiraterone/enzalutamide)	0.64 (0.46–0.89)	0.0086
M1 disease	1.37 (0.96–1.95)	0.0824
Visceral metastases	1.34 (0.89–2.02)	0.1552
Gleason score 8–10 at diagnosis	1.16 (0.81–1.67)	0.4113
Prior therapy with curative intent	0.87 (0.59–1.28)	0.4740
Type of progression (radiologic vs PSA only)	0.95 (0.44–2.03)	0.8860
Type of progression (pain status at study entry vs PSA only)	1.80 (0.94–3.43)	0.0740
Hemoglobin, g/L	0.98 (0.96–0.99)	0.0002
LDH, IU/L (log_{10})	3.75 (1.69–8.32)	0.0012
Alkaline phosphatase, IU/L (log_{10})	1.97 (1.24–3.12)	0.0038
PSA, ng/mL (log_{10})	1.69 (1.31–2.18)	< 0.0001
Duration of first ADT, months	0.99 (0.98–1.00)	0.0582
NLR, per 1-unit increase	1.04 (1.02–1.07)	< 0.0001
Neutrophil count, 10^9/L	1.10 (1.01–1.20)	0.0267
Age, years	1.00 (0.98–1.02)	0.8444
Testosterone, nmol/L	0.89 (0.58–1.38)	0.6046

Notes: Univariate Cox regression model stratified for given stratification factors adjusted for treatment and each baseline risk factor.

Stratification factors were ECOG performance status (0–1 vs 2), time from ARTA initiation to progression (0–6 months vs 6–12 months), and timing of the ARTA (before vs after docetaxel), as specified at the time of randomization.

A log_{10} transformation was used for PSA, LDH, and alkaline phosphatase.

Categorical factors: M1 disease, visceral metastases, Gleason score 8–10 at diagnosis, prior therapy with curative intent, and type of progression.

Continuous factors: Hemoglobin, LDH, alkaline phosphatase, PSA, duration of first ADT, NLR, neutrophil count, age, and testosterone.

Abbreviations: ADT, androgen deprivation therapy; ARTA, androgen-receptor-targeted agent; CI, confidence interval; ECOG, Eastern Cooperative Oncology Group; HR, hazard ratio; LDH, lactate dehydrogenase; NLR, neutrophil-to-lymphocyte ratio; OS, overall survival; PSA, prostate-specific antigen.

Table A.3. Multivariate Cox regression analysis of OS: stepwise selection model

**Baseline risk factor**	**HR (95% CI)**	***P* value**
Treatment (cabazitaxel)	0.63 (0.42–0.94)	0.0222
NLR, per 1-unit increase	1.05 (1.02–1.08)	0.0003
Hemoglobin, g/L	0.98 (0.96–0.99)	0.0051
PSA, ng/mL (log_{10})	1.54 (1.11–2.14)	0.0108

Abbreviations: CI, confidence interval; HR, hazard ratio; NLR, neutrophil-to-lymphocyte ratio; OS, overall survival; PSA, prostate-specific antigen.
